# The prevalence of Epilepsy and its co-occurrence with alcohol dependence among polish prisoners

**DOI:** 10.1186/s12939-019-1009-z

**Published:** 2019-07-02

**Authors:** Barbara Stawińska-Witoszyńska, Katarzyna Czechowska, Barbara Więckowska

**Affiliations:** 1 Department of Epidemiology and Hygiene, Chair of Social Medicine, Poznan University of Medical Sciences, Rokietnicka 4, 60-806 Poznan, Poland; 2 Department of Computer Science and Statistics, Poznan University of Medical Sciences, Poznan, Poland

**Keywords:** Epilepsy, Alcohol dependence, Prisoners

## Abstract

**Background:**

For a large part of the prisoners population, the prevalence of many diseases and the number of risk factors are greater than for the general population. In this work, we present an analysis of the prevalence of epilepsy and its co-occurrence with alcohol dependence among prisoners in a Polish penitentiary.

**Methods:**

One and multidimensional logistic regression was used to present the relationship between epilepsy and the co-occurrence of alcohol dependence and of other variables like: the prisoners’ age, their classification, the unit type, the length of the stay in the penitentiary, and professional activity.

**Results:**

More than 7% of the prisoners had epilepsy. The prevalence was significantly higher in the 40–49 age group and among prisoners aged 50 and older. For prisoners suffering from alcohol dependence, the probability of epilepsy was over four times higher than for prisoners who did not suffer from that condition (OR [95%CI] = 4.09 [1.82–9.17], *p* = 0.001].

**Conclusions:**

The obtained results show that the prevalence of epilepsy and alcohol dependence in the studied prisoner population was much higher than in the general population of Poles and that alcohol dependence was strongly correlated with epilepsy, independent from other variables. The research allowed to assess the size of the analyzed problem among convicts, however, bearing in mind the multifactorial etiology of epilepsy, the cause and effect relationship between exposure to alcohol and its occurrence would require further in-depth analytical studies taking into account other etiological factors of this disease.

## Introduction

Because of the huge number of prisoners requiring treatment, the recommendations of Resolution on research on prisoners considered from the individual angle and on the prison community put an emphasis on the need to conduct research within the prison community. The results of studies on the treatment of somatic diseases, personality disorders, and various dependencies show that about 25% of prisoners in Poland require treatment [1].

Individual medical examinations and psychological evaluations, including the mandatory ones, as well as scientific research conducted among prisoners, must be carried out in accordance with the appropriate provisions of executive penal code [2]. After the European Union standards which unify the standards of serving a sentence of imprisonment in the member states have been implemented in Poland, and after the information technology has been developed, we know more about prisoners’ health, however, that knowledge is still insufficient [3]. The studies conducted in the prisoner population focus on addictions and psychological diseases and disorders more often than on the prevalence of other diseases [4–6]. In this work, we present a part of the results of epidemiological studies on the health of prisoners from a penitentiary in Poland, taking into account chronic non-communicable diseases and communicable diseases, in particular, the prevalence of epilepsy and its co-occurrence with alcohol dependence. We discuss the results of epidemiological studies carried out in a penitentiary in Poland which concern the prevalence of epilepsy and its co-occurrence with alcohol dependence.

### The aim of this work

The aim of this work was to determine the prevalence of epilepsy in the prisoner population and of its co-occurrence with alcohol dependence.

## Material and methods

For the descriptive epidemiological study, we used secondary information sources and the medical files of all 635 prisoners aged 21–72 from a Polish penitentiary for men serving a sentence of imprisonment for the first time and for penal recidivists. That penitentiary encompasses three types of units: closed, half-open, and open, which differ with regard to the manner in which the residential buildings are secured and to prisoner duties.

The material for the analysis were the prisoners’ health books, which contained the results of specialist consultations and of additional examinations, and their medical files from the time before their imprisonment. In the penitentiary, the prisoners’ epilepsy was diagnosed by a neurologist, and alcohol dependence – by a psychiatrist in the same way not imprisoned polish patients are being diagnosed.

In the diagnostic procedure of epilepsy additionally to basic tests - blood tests, ECG and electroencephalographic tests, magnetic resonance and computer tomography was performed that currently is being recognized as the method of choice in diagnosis of the disease [7]. CT should be urgently performed in case of patients that have a threat of occuring seizures, eg first time occurred epileptic seizure due to withdrawal syndrome or if there are any contraindications to undergo magnetic resonance imaging [8].

The alcohol dependency syndrome was diagnosed on the basis of diagnostic criteria contained in the Classification of Mental Disorders and ICD-10 Behavior Disorders and screening tests, in case of a correctional facility it was a diagnostic questionnaire alcohol dependence syndrome according to dr. Woronowicz [9, 10].

In the analysis of the prevalence of epilepsy, we took into account the following variables: the prisoners’ age, the prisoners’ classification (first-time, recidivist), the unit type (closed, half-open and open), the length of the stay in the penitentiary (less than a year, from one to 5 years, more than 5 years). We also determined the time of the diagnosis of epilepsy (before or during the stay in the penitentiary) and its co-occurrence with alcohol dependence.

The statistical analysis of the occurrence of epilepsy and alcohol dependence was carried out in two stages. First, we determined the influence of such factors as: the occurrence of alcohol dependence, the prisoners’ age, their classification, the unit type, the length of the stay in the penitentiary, and professional activity on the prevalence of epilepsy, with the use of the univariate logistic regression model, and we provided the odds ratio together with the 95% confidence interval.

At the second stage, in order to determine the independent influence of alcohol dependence on the occurrence of epilepsy, regardless of other characteristics of the prisoners, we adjusted the odds ratio obtained during the first stage. We began by adjusting the odds ratio based on those variables which had a statistically significant influence on the occurrence of epilepsy in the univariate logistic regression model (minimally adjusted model), and then we proceeded to adjust it based on all analyzed variables (fully adjusted model).

We adopted 0.5 significance level. The analyses were made with the use of the PQStat v1.6.6 program.

## Results

Forty-five of the 635 prisoners, that is, 7.1% of them were diagnosed with epilepsy. In most cases (86.7%), the diagnosis had been made before the men were imprisoned. Epilepsy was the most common in the 40–49 age group and among those prisoners who were 50 and older (10.9 and 10.8% of the prisoners in those age groups, respectively). In both age groups, epilepsy was significantly more common than among prisoners younger than 30. (Table 1).

**Table 1:** The prevalence of epilepsy depending on the occurrence of alcohol dependence and of other variables

	Epilepsy			Logistic regression model^a^		
Variables	yes	no	% of ill people	*p* value	OR [95%CI]	
	*n* = 45	*n* = 590				
Age						
younger than 30	9	196	4.40%	reference category		
30–39	14	213	6.20%	0.413	1.43 [0.61–3.38]	
40–49	12	98	10.90%	0.032	2.67 [1.09–6.54]	
50 years and older	10	83	10.80%	0.043	2.62 [1.03–6.69]	
The length of the imprisonment by the time of the study						
up to 1 year	12	194	5.80%	reference category		
1–5	29	320	8.30%	0.282	1.47 [0.73–2.94]	
more than 5 years	4	76	5.00%	0.785	0.85 [0.27–2.72]	
Classification						
first-time prisoner	20	357	5.30%	reference category		
recidivist	25	233	9.70%	0.037	1.92 [1.04–3.53]	
Unit type						
closed	22	233	8.60%	0.217	1.47 [0.8–2.69]	
open and half-open	23	357	6.10%	reference category		
Professional activity						
yes	16	306	5.00%	0.038	0.51 [0.27–0.96]	
no	29	284	9.30%	reference category		
Alcohol dependence						
yes	9	34	20.90%	0.001	4.09 [1.82–9.17]	
no	36	556	6.10%	reference category		

^a^univariate model (without adjustment)

For prisoners aged 40–49 and those aged 50 and older, the probability of epilepsy was almost twice as high as for those younger than 30 (OR [95%CI] = 2.67 [1.09–6.54], *p* = 0.032), (OR [95%CI] = 2.62 [1.03–6.69], *p* = 0.043) (Table 1).

Epilepsy was more common among recidivists than among the men who served a sentence of imprisonment for the first time. That difference was statistically significant (*p* = 0.037). For recidivists, the probability of epilepsy was almost twice as high as for the other group (OR [95%CI] = 1,92 [1,04–3.53], *p* = 0,037) (Table 1).

The men who had been imprisoned for 1–5 years were the group with the highest prevalence of epilepsy, however, there were no statistically significant differences in the prevalence of epilepsy among the groups of prisoners with varying lengths of stay in the penitentiary. The differences were also statistically insignificant (*p* > 0.05) for various unit types: closed versus open and half-open (the open and half-open types were combined because of the small number of prisoners in the open-type unit). The prevalence of epilepsy in those two groups was 8.6 and 6.1, respectively (Table 1).

Unsurprisingly, the number and percentage of men with diagnosed epilepsy among those who did not work and those who did differed. Twenty-nine prisoners who did not work (9.3% of that group) had the disease. In the group of working prisoners, 16 (5%) had it.

The probability of epilepsy among professionally active prisoners was almost two times lower than among the professionally inactive ones (OR [95%CI] = 0.51 [0.27–0.96], *p* = 0,037), which can be explained by the fact that prisoners with milder cases of epilepsy could work while those who were more severely ill were not qualified for professional activity by their physician.

Alcohol dependence was diagnosed in 43 men (6.8% of the prisoners). In 26 cases, that diagnosis was made in the penitentiary. From the prisoners with diagnosed alcohol dependence, 20.9% had epilepsy. In the prisoner group without alcohol dependence, that percentage was 6.1%.

For prisoners suffering from alcohol dependence, the probability of epilepsy was over four times higher than for prisoners who did not suffer from that condition (OR [95%CI] = 4.09 [1.82–9.17], *p* = 0.001] (Table 2).

**Table 2:** The correlation between alcohol dependence and the occurrence of epilepsy in prisoners, after adjustment for other potential risk factors

	Logistic regression model			
	Minimally adjusted^a^		Fully adjusted^b^	
Alcohol dependence	*p* value	OR [95%CI]	*p* value	OR [95%CI]
no	reference category		reference category	
yes	0.008	3.17 [1.36–7.39]	0.017	2.86 [1.2–6.81]

^a^adjustment for age, classification, and professional activity

^b^adjustment for age, classification, and professional activity, the length of imprisonment, and the unit type

High correlation between epilepsy and the co-occurrence of alcohol dependence in prisoners was observed regardless of the presence of other risk factors for that disease (Table 2). In the model minimally adjusted for the significant variables related to the occurrence of epilepsy (age, prisoner classification, and professional activity) and in the fully adjusted model (adjustment for age, classification, and professional activity, the length of imprisonment, and the unit type), the odds ratio was, respectively (OR [95%CI] = 3.17 [1.36–7.39], *p* = 0.008 and OR [95%CI] = 2.86 [1.2–6.81].

## Discussion

Epilepsy is one of the most common neurological diseases. It is defined as “a neurological disorder marked by sudden recurrent episodes of sensory disturbance, loss of consciousness, or convulsions, associated with abnormal electrical activity in the brain” [11]. The number of people suffering from that disease in the world is estimated to be about 50 million (in Europe – 6 million) [12–14]. The prevalence of epilepsy in Poland in recent years was evaluated to be similar: about 1% of the population, that is, about 300–400 thousand people [15–17]. It is estimated that prisoners suffer from epilepsy twice as often as the general population. A meta-analysis of data from 1966 to 2001, encompassing seven studies conducted in the United States of America, the United Kingdom, and Nigeria, in the prisoner population, mainly men (90%) aged, on average, 29, indicated a 0.7%prevalence of epilepsy. The results could have been influenced by the methodology of the studies which were only based on clinical interviews carried out by means of surveys and by the subjects’ age, that is, by the lack of the elderly who are the second group – after children – with the highest incidence of epilepsy [18]. In studies carried out in prisons in Canada in 1993–2013, the number of prisoners with epilepsy was estimated to be 1–4% of that population [19]. The results of studies conducted in France among 1221 prisoners showed a 5.9% prevalence of epilepsy. That was a lower level than the one in the prisoner population studied in the penitentiary in Poland [20].

Based on the surveys of Turkish prisoners diagnosed with epilepsy it was found, that the most common mental disorders coexisting with epilepsy was depression and anxiety disorders that occurred with the same frequency as among people from the general population with epilepsy. In contrast, personality disorders, addiction to psychoactive substances and bipolar disorder were more common among prisoners with diagnosed epilepsy [21]. The subject of our research was not the coexistence of epilepsy with other neurological diseases and mental disorders, only the frequency of occurrence, in addition to epilepsy and alcohol dependency syndrome, personality disorders, anxiety disorders and depression. Disorders qualified for the group of neurological disorders and mental disorders were the most commonly diagnosed abnormalities in the prisoners population, however, the percentage of depressed patients turned out to be lower compared to the overall Polish population. This last fact can be important in the context of cross-sectional American research among prisoners, published in 2017 reporting an increased chance of a suicide attempt convicts with bipolar disorders and anxiety disorders, then addiction to alcohol and depression, and addiction to marijuana and depression [22].

The Commission on Classification and Terminology of the International League Against Epilepsy has proposed an etiological classification of epilepsy into three subtypes: structural-metabolic, genetic, and of unknown etiology. In the case of nearly 65–70% of people with epilepsy, the etiology of that disease remains unknown. One recognized cause of epilepsy are lesions of vascular origin in the brain, especially of elderly people. Some other recognized causes are: injuries, brain tumors, neurodegenerative brain diseases, and infections of the central nervous system. The degree to which toxic factors and some metabolic disorders play a role in the etiology of epilepsy is estimated to be 1,5% [23, 24]. It has been noted that it is sometimes difficult to distinguish between the overlapping etiological factors and the triggering factors (such as temporary electrolyte disturbances) [25].

In the literature on the subject, we can find information about frequent epileptic seizures among prisoners and about the insufficient number of diagnostic examinations. Fewer than 20% of prisoners undergo diagnostic examinations in the form of computer tomography, and virtually no MRI scans are done. For that reason, traumatic brain injury, which plays an important role in the etiology of epilepsy, is not diagnosed. In the studied population, in 2017 six tomography, 5 electroencephalograms and one Magnetic resonance examination were performed.

The interviews conducted with the subjects in the penitentiary in Poland, especially from the younger age groups, contained information about serious car accidents or batteries which resulted in a loss of consciousness. According to Plech, head injuries may be the cause of 20% epilepsy cases [26].

Unfortunately, post-traumatic epilepsy often remained undiagnosed because of incomplete medical files and the lack of diagnostic examinations. The diagnostic problems were compounded by the presence of other risk factors for that disease, including alcohol dependence. Many subjects with diagnosed epilepsy declared simultaneous alcohol abuse and having been in rehabilitation centers. Many prisoners from that penitentiary were from dysfunctional, impoverished, and poorly educated families. Such an environment was conducive to alcohol abuse. The collected data confirm that alcohol dependence was more prevalent among the studied prisoners than among the general population of Poland. According to Maciej Frąckowiak and Marek Motyka, 2% of the Polish society is alcohol dependent [27]. Among the prisoners, the prevalence of alcohol dependence was 6.8%. That is why we decided, in our work, to take into account the co-occurrence of alcohol dependence with epilepsy. The results of our research confirm that epilepsy was over four times as frequent in people with diagnosed alcohol dependence than in the remaining group of prisoners, which is consistent with the results of Danish studies which showed that the probability of epilepsy was higher in people with alcoholic liver cirrhosis (OR = 3.74, 95% CI 3.56–3.94) [28]. We have determined a high, independent correlation between epilepsy and alcohol dependence among the studied Polish prisoners, both in the model which was minimally adjusted for statistically significant variables related to the occurrence of epilepsy (that is, age, prisoner classification, and professional activity) and in the fully adjusted model. The conclusion is that greater prevalence of epilepsy in prisoners with alcohol dependence was independent of other characteristics of those men. Nevertheless, we recognize that our study has a limitation stem from the nature of observational data utilized in our study. It is difficult for us to give the exact time span between the start of alcohol consumption and the epilepsy occurrence of the inmates Due to the difficulty of detecting diagnostic date, the study was not able to examine the temporal relationship between epilepsy and alcohol dependence.

Moreover, based on descriptive epidemiological studies, it is difficult to determine the exact type of epileptic seizures in the studied population.

The chronic consumption of alcohol alone, without the coexistence of additional diseases, can increase the risk of the first epileptic seizure 2–10 times, depending on the amount of its consumption per day and the frequency of seizures in the case of the abstinence syndrome is estimated depending on the study from 28 to 88% [29]. Among those addicted to alcohol with already diagnosed idiopathic or symptomatic epilepsy may also occur withdrawal syndrome or arise seizures that are the consequences of chronic, toxic exposure of the central nervous system for alcohol, causing changes in the cellular level (alcoholic epilepsy). In case of people with alcohol dependency syndrome, it is not only more common already mentioned head injuries, but high alcohol consumption increases their risk of strokes [29]. The risk of epilepsy as a consequence of brain damage after injuries and accidents can occur even many years after those events [30].

The epileptic seizures which took place in the penitentiary frequently happened to people who had just arrived there and were still in the dispensary or reception room, before they were admitted to a prison cell. In statistics were included only diagnosed cases of epilepsy, not isolated epileptic seizures. Which can occur to anyone, including abstinent syndrome, that does not decide about having this disease. Some prisoners with epilepsy which had been diagnosed before their imprisonment came to the penitentiary after they had been neglecting their health during many days of continuous alcohol abuse, and admitted that they had stopped treatment. The immediate introduction of pharmacological treatment did not always prevent seizures which usually appeared within 3 days from the admission to the penitentiary. Not only the health care employees, but also civil employees and functionaries are well-prepared for the possibility of the occurrence of seizures and know the procedures of conduct. The rescue workers who work in the dispensary periodically train other employees as well as the prisoners in first aid.

Because the prisoners arrived to the prison mostly with the diagnosis and implemented pharmacological treatment medical therapy was mainly being continued during their stay in penitentiary, but in justified cases further diagnosis was carried out at the request of the physician. For the substance abuse treatment, the convicts in the chronic phase of alcoholism go over the psychotherapy program of addiction with rehabilitation and resocialization carried out in the prison therapeutic departments. Only some prisoners want to get out of the addiction and expect help. The motives of others are related to improving the conditions of the sentence, chance for early, conditional release or recovery of health for further criminal activity, which is limited by the alcohol problem. Because many convicts come from pathological environments, without further support after the release they can quickly return to addiction [31].

## Conclusion

The obtained results show that the prevalence of epilepsy and alcohol dependence in the studied prisoner population was much higher than in the general population of Poles and that alcohol dependence was strongly correlated with epilepsy, independent from other variables. The research allowed to assess the size of the analyzed problem among convicts, however, bearing in mind the multifactorial etiology of epilepsy, the cause and effect relationship between exposure to alcohol and its occurrence would require further in-depth analytical studies taking into account other etiological factors of this disease.

## Data Availability

Data and materials used for the manuscript were taken from medical documentation of prison clinic and prison registration data after obtaining written consent of prison authorities.

## Abbreviations

CT: Computed tomography
ECG: Electrocardiogram
EEG: Electroencephalogram
MRI: Magnetic resonance imaging
